# Daidzein ameliorates peripheral neuropathy in Sprague Dawley rats

**DOI:** 10.3389/fphar.2024.1385419

**Published:** 2024-08-06

**Authors:** Ankit P. Laddha, Yogesh A. Kulkarni

**Affiliations:** Shobhaben Pratapbhai Patel School of Pharmacy & Technology Management, SVKM’s NMIMS, Mumbai, India

**Keywords:** daidzein, diabetic neuropathy, NADPH oxidase, streptozotocin, diabeteic complication

## Abstract

Neuropathy is the most common disorder comprising peripheral nerve damage in diabetic patients. Prolonged hyperglycaemia and oxidative stress cause metabolic imbalance and are the key reasons for the development of diabetic neuropathy. Daidzein, a soy isoflavone possesses potent anti-hyperglycaemic and antioxidant activity. The present study aims to check the protective effect of Daidzein in diabetic neuropathy in rats. The experimental animal model involved induction of diabetes in rats by intraperitoneal injection of streptozotocin (55 mg/kg). Following confirmation of diabetes, the diabetic rats were subjected to oral treatment with varying doses of Daidzein (25, 50, and 100 mg/kg) and pregabalin (30 mg/kg) for a duration of 4 weeks, initiated 6 weeks after diabetes induction. Results indicated that Daidzein treatment led to a significant reduction in plasma glucose levels and an improvement in body weight among diabetic animals. Moreover, Daidzein demonstrated a positive impact on sensory functions, as evidenced by the effect on tail withdrawal and response latency. Mechanical hyperalgesia and allodynia, common symptoms of diabetic neuropathy, were also significantly reduced with both Daidzein and pregabalin treatment. Notably, nerve conduction velocities exhibited improvement following the administration of Daidzein and pregabalin. Further investigation into the molecular mechanisms revealed that Daidzein treatment resulted in a notable enhancement of antioxidant enzyme levels and a reduction in the overexpression of NOX-4 in the sciatic nerve. This suggests that Daidzein’s therapeutic effect is associated with the inhibition of oxidative stress via NOX-4. In summary, the findings of study suggests that, Daidzein treatment significantly attenuated diabetic neuropathy by inhibiting oxidative stress via NOX-4 inhibition.

## 1 Introduction

Diabetes and associated complications have become important causes of mortality these days. A prolonged increase in blood glucose level due to pancreatic β cell damage or insulin resistance is the crucial reason for the development of abnormal metabolic, humoral, and hemodynamic functions (Domingueti et al., 2016).

Diabetic neuropathy is the most prevalent and painful complication of Diabetes and is considered the most deceptive and less-understood complication (Schreiber et al., 2015). It comes under the third most common neurological disorder, which includes approximately 30% of hospitalized diabetic patients and 20%–30% of community-based people (Dewanjee et al., 2018). Initially, the chances of developing peripheral neuropathy in adults with type-I Diabetes are 6%, increasing by 30% after 13–14 years of persistent hyperglycaemia (Schreiber et al., 2015).

Diabetic neuropathy is a broad term that includes various types of neuropathies depending on the damaged nerve type. Diabetic peripheral neuropathy affects all peripheral nerves, including pain fibers. Numbness and pain are the most common, and foot amputation and loss of sensation are the least common symptoms reported in peripheral neuropathy. Prolonged hyperglycaemia develops oxidative stress and causes alternation in neuro-immune interactions, neural and glial cell apoptosis, and inflammation. Increased oxidative stress in hyperglycaemia is mainly due to auto-oxidative glycosylation, advanced glycation end products (AGEs) formation, and sorbitol deposition due to the polyol pathway activation (Nasiry et al., 2017). These pathways trigger neuroinflammation and neuronal damage by activation of NADPH oxidase (NOX), which further leads to the development of inflammatory markers like nuclear factor-kappa beta (NF-kβ), p38 mitogen-activated protein kinases (p38MAPK), cyclooxygenase-2 (COX-2), 12/15-lipoxygenase and oxidative stress enzyme-like PARP (Hosseini and Abdollahi, 2013). NOX isoform, NOX-4 is an important source of reactive oxygen species (ROS) in somatic cells. It is also widely expressed in a subset of nonpeptidergic nociceptors, including astrocytes, microglia, vascular endothelial cells, and myelinated dorsal root of ganglia, contributing to medicate pain sensitization (Volpe et al., 2018). Recent evidence identified that NOX-4 is an early initiator of neuropathic pain (Geis et al., 2017). Sensitization because of persistent pain is because of the formation of reactive oxygen species such as hydrogen peroxide (H_2_O_2_) and superoxide (O_2_^−^) and the active superoxide by-product peroxynitrite due to NOX-4 activation in sciatic nerve (Rayegan et al., 2017).

NOX-4 is a membrane-bound enzyme present in a dissociated form in a resting state and becomes assembled into a functional oxidase complex upon stimulation and generates superoxide free radical by transferring one electron to oxygen from NADPH during prolonged hyperglycaemia.

Daidzein is a naturally occurring compound mainly present in legumes, especially soybeans. It belongs to the isoflavone class and is commonly known as soy isoflavone. Daidzein is reported to have beneficial effects in the treatment of cardiovascular diseases (Kim et al., 2009), neurodegenerative diseases (Wei et al., 2019), cancer (Zheng et al., 2017) and diabetes (Das et al., 2018). Daidzein shows a beneficial effect against hyperglycemia by improving impaired glucose and lipid metabolism (Das et al., 2018). It improves insulin sensitivity by activating peroxisome proliferator-activated receptor gamma (PPAR-γ), enhances antioxidant defenses, and exerts anti-inflammatory effects by inhibiting pro-inflammatory cytokines (Chen et al., 2016). Daidzein also modulates glucose metabolism by increasing glucokinase activity and decreasing glucose-6-phosphatase activity, and it improves lipid profiles by reducing triglycerides and LDL cholesterol while increasing HDL cholesterol. Literature reported on Daidzein also reveals that it possesses potent antioxidant and anti-inflammatory activity (Peng et al., 2017) and also prevented the progression of diabetic complications like diabetic retinopathy (Laddha and Kulkarni, 2021a) diabetic cardiomyopathy (Laddha and Kulkarni, 2021b), and diabetic cytopathic (Laddha and Kulkarni, 2022) by inhibiting NADPH oxidase enzyme activity which is involved in the generation of ROS (Elumalai et al., 2021).

Oxidative stress mediated by NADPH oxidase (NOX-4) plays a crucial role in diabetic peripheral neuropathy. However, there are no existing reports on the effects of daidzein on diabetic neuropathy through NOX-4 inhibition. Therefore, this study was designed to investigate the effects of daidzein on diabetic neuropathy and to examine the involvement of NOX-4 in oxidative neuronal damage under diabetic conditions.

## 2 Materials Methods

### 2.1 Experimental animals

Animal experimentation was carried out as per the NIH guideline for the care and use of laboratory animals (NIH Publication No. 80–23; revised 1978) 200–220 g male *Sprague Dawley* rats were used and were procured from the National Institute of Biosciences, Pune, India.

### 2.2 Experimental design

After 1 week of acclimatization, diabetes was induced in rats via intraperitoneal injection of streptozotocin (Sigma Aldrich, St. Louis, United States) at a dose of 55 mg/kg. Plasma glucose was determined after 7 days of streptozotocin (STZ) administration. Animals with plasma glucose 250 mg/dL were considered diabetic and selected for study, animals were randomized into six groups (n = 10, each group) depending on their body weight and plasma glucose and were treated for the next 4 weeks (Suryavanshi and Kulkarni, 2020).

Group 2 was considered a diabetic control group; animals in this group were left untreated and received 0.5% of sodium carboxymethylcellulose (CMC). Group 3 was considered as the standard drug treatment group; animals in this group received pregabalin orally at a dose of 30 mg/kg. Groups 4, 5, and 6 were considered treatment groups; animals in these groups received Daidzein (Combi-Blocks, Inc. San Diego, United States) orally at doses of 25, 50, and 100 mg/kg. Group 1 was considered a normal control group and contained non-diabetic animals. Animals in this group received 0.5% of CMC as a vehicle.

### 2.3 Evaluation parameters

#### 2.3.1 Determination of body weight and plasma glucose

Animals were weighed after 28 days of treatment and blood was withdrawn from the retro-orbital plexus and collected in a microcentrifuge tube containing 20 μL of disodium ethylenediaminetetraacetic acid (EDTA) as an anticoagulant. Plasma was separated after centrifugation and used for the determination of glucose using GOD-POD kit (Transasia Biomedicals Ltd., India).

#### 2.3.2 Behavioural assessment for heat nociception

At the end of the treatment, the latency to the heat stimuli was used to evaluate the heat nociception threshold by using a hot plate and tail immersion method.

In the tail immersion test, animals were restrained for 30 min before the test. The tail of each rat from a different treatment group was immersed in a water bath maintained at 55°C ± 1°C until tail withdrawal or sign of struggle was observed. The cut-off time was kept at 15 s to avoid injury. The reaction time i.e., time required to withdraw the tail from hot water, was recorded.

In Eddy’s hot plate test, each animal was kept on hot plate apparatus (IITC Life Science, United States) maintained at 55°C ± 0.5°C. The latency to the first response in the form of flickering, licking of the hind paw, or jumping was recorded. The cut-off time was kept at 15 s to avoid damage to the paw (Oza and Kulkarni, 2020).

#### 2.3.3 Behavioural assessment for mechanical hyperalgesia

Mechanical hyperalgesia nociceptive threshold was determined using the Randall - Selitto test. After 28 days of treatment, animals were restrained and received increasing pressure stimulus with a pressure applicator (IITC Life Science, United States) to the dorsal surface of the hind paw. The maximum pressure where animals try to withdraw their paw was recorded with Randall Selitto paw pressure test apparatus (IITC Life Science, United States). The same was performed three times at an interval of 20 min and the average reading was considered. The cut-off time was kept at 600 g to avoid damage because of excessive pressure (Kayser, 2013).

#### 2.3.4 Behavioural assessment for mechanical allodynia

The nociceptive threshold to the innocuous stimuli was determined using Von-Frey filament. Animals after treatment were kept in the elevated cage, which contains wire mesh at the bottom. Animals were acclimatized in the cage for 20 min. A constantly increasing pressure stimulus was applied to the ventral surface of the hind paw with an electronic Von Frey rigid tip (IITC Life Science, United States). The pressure at which animals showed responses like paw lifting and withdrawal was recorded with the help of electronic Von Frey apparatus (IITC Life Science, United States). Readings were recorded thrice after at least 20 min intervals. The cut-off time was kept at 400 g to avoid damage because of excessive pressure (Ängeby Möller et al., 1998).

#### 2.3.5 Determination of nerve conduction velocity

Nerve conduction velocities, motor nerve conduction velocity (MNCV), and sensory nerve conduction velocity (SNCV) were recorded from the sciatic nerve using a data acquisition system Power Lab (AD Instruments, Australia). After 28 days of treatment, animals were anesthetized using urethane (1.2 g/kg, *i.p.*). The rectal temperature of animals was continuously monitored using a homeothermic blanket with a flexible probe (230 VAC, Harvard Apparatus, United States) and was maintained at 37°C ± 0.5°C. A 24 gauge needle electrode having a single 5 V stimulus was used to stimulate the sciatic nerve from the sciatic notch and tibial nerve (proximal to ankle) region. Receiving electrodes (+ve, −ve, and earth) were placed in the foot muscle. The “M” wave and “H” wave refluxes were recorded digitally using a data acquisition system-poerlab (AD Instruments, Australia). The distance between the stimulation and receiving point was also measured manually using a ruler (Bhatt and Veeranjaneyulu, 2010). MNCV and SNCV were recorded from “M” wave and “H” wave latency using the following formula - MNCV/SNCV (m/s) = (Distance between stimulating and recording electrode)/latency.

#### 2.3.6 Determination of oxidative stress parameters

At the end of the study, animals were humanely sacrificed, and the sciatic nerve from the left side of the posture was isolated and homogenized in 0.1 M phosphate buffer (pH 7.4) using a probe homogenizer (Polytron PT 2500E, Kinematica, Switzerland). The protein concentration was determined in tissue homogenate with the method described earlier (Lowry et al., 1951). Homogenate was used for the determination of levels of reduced glutathione (GSH), and malondialdehyde (MDA) according to the standard protocol mentioned in the literature (Ellman, 1959; Ohkawa et al., 1979). Some part of the homogenate was centrifuged at 2500 *g* and 10,000 rpm for 10 min and 20 min, respectively, for separation of a post-nuclear and post-mitochondrial fraction, which were used for the determination of catalase (CAT) and superoxide dismutase (SOD) activity (Paoletti et al., 1990).

#### 2.3.7 Western blot analysis

Expression of NOX-4 in the sciatic nerve of the different treatment groups was determined using NOX-4 (rabbit; 1:500) (ABclonal Technology, United States) primary antibody and peroxidase-labeled goat anti-rabbit IgG secondary antibody. The quantification was carried out as per the previously reported method by Oza and Kulkarni (Oza and Kulkarni, 2020).

The results were expressed as relative expression i.e., NOX-4/β-actin using Image Studio Lite Ver 5.2 software.

## 3 Statistical analysis

One-way ANOVA followed by Bonferroni compared selected pairs of column tests were performed using Graph pad Prism 6 software. All the data were expressed in Mean ± SD.

## 4 Results

### 4.1 Effect of daidzein on body weight and plasma glucose

The diabetic control group showed a significant (*p* < 0.001) decrease in body weight compared to normal control animals. Treatment with Daidzein at doses of 50 and 100 mg/kg significantly (*p* < 0.05, *p* < 0.001) improved the body weight compared to diabetic control animals. Daidzein 50 mg/kg dose showed 31.53% and 100 mg/kg dose showed 45.2% improvement in body weight whereas pregabalin treatment showed 30.74% improvement. Treatment with pregabalin also significantly (*p* < 0.05) improved body weight.

**FIGURE 1:**
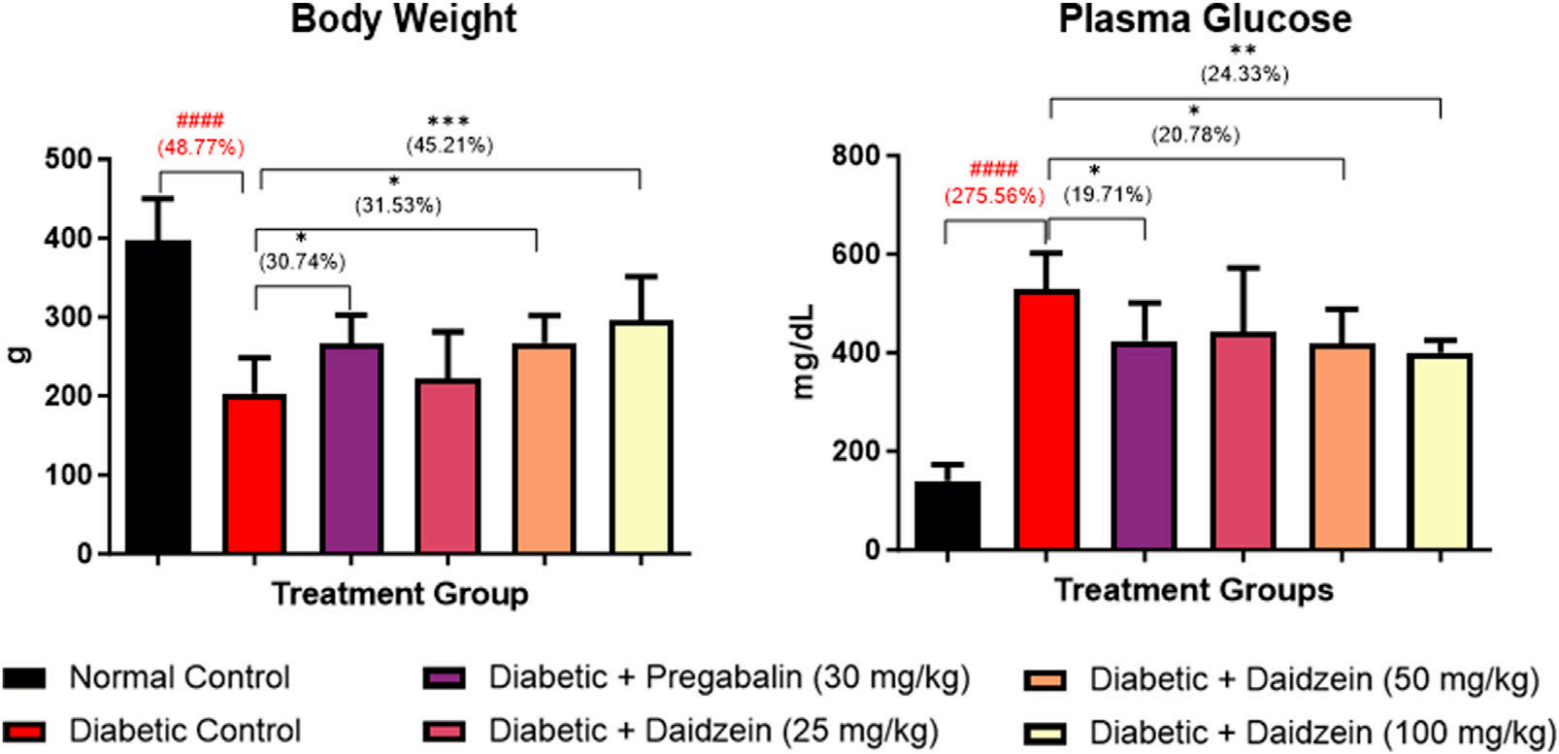
Effect of Daidzein on body weight and plasma glucose levels. All values are expressed as Mean ± SD. ***p < 0.001, **p < 0.01, *p < 0.05 indicates the level of significance when treatment groups compared with the diabetic control group and ^###^ *p* < 0.001 when the diabetic control group compared with the normal control group.

A significant increase in glucose level (*p* < 0.001) was observed in diabetic control animals compared to normal control animals. Daidzein treatment at doses of 50 and 100 mg/kg and pregabalin at a dose of 30 mg/kg significantly reduced (*p* < 0.05, *p* < 0.01, and *p* < 0.05) the elevated level of blood glucose when compared with diabetic control animals. There is 20.78% and 24.33% reduction in blood glucose in 50 mg/kg and 100 mg/kg doses of daidzein respectively and 19.71% by pregabalin treatment (Figure 1).

### 4.2 Effect of daidzein on behavioural parameters

Significant decreases in tail withdrawal latency (*p* < 0.001) and response latency (*p* < 0.001) were observed in diabetic control animals during tail immersion and hot plate tests as compared to normal control animals. Treatment with Daidzein at a dose of 100 mg/kg significantly improved the tail withdrawal latency (*p* < 0.001) compared to disease control animals after 28 days of treatment. Improvement in tail withdrawal latency is 108.82%. Daidzein at a dose of 50 and 100 mg/kg and pregabalin (30 mg/kg) significantly improved (*p* < 0.05, *p* < 0.01, *p* < 0.05) the response latency after 4 weeks of treatment. Response latency is increased by 52.4% and 53.55% by Daidzein 50 mg/kg and 100 mg/kg doses and 46.06% by pregabalin treatment.

Diabetic control animals also showed a significant decrease in paw withdrawal latency (*p* < 0.001) measured using a Randall – Selitto test (Mechanical hyperalgesia) when compared to normal control animals. Treatment with Daidzein at a dose of 50 and 100 mg/kg and pregabalin (30 mg/kg) significantly improved (*p* < 0.05, *p* < 0.01 *p* < 0.05) the paw withdrawal latency. Withdrawal latency in Randall-Selitto test was improved by 86.07% and 112.01% by mid and high doses of daidzein and 90.28% by pregabalin treatment.

**FIGURE 2:**
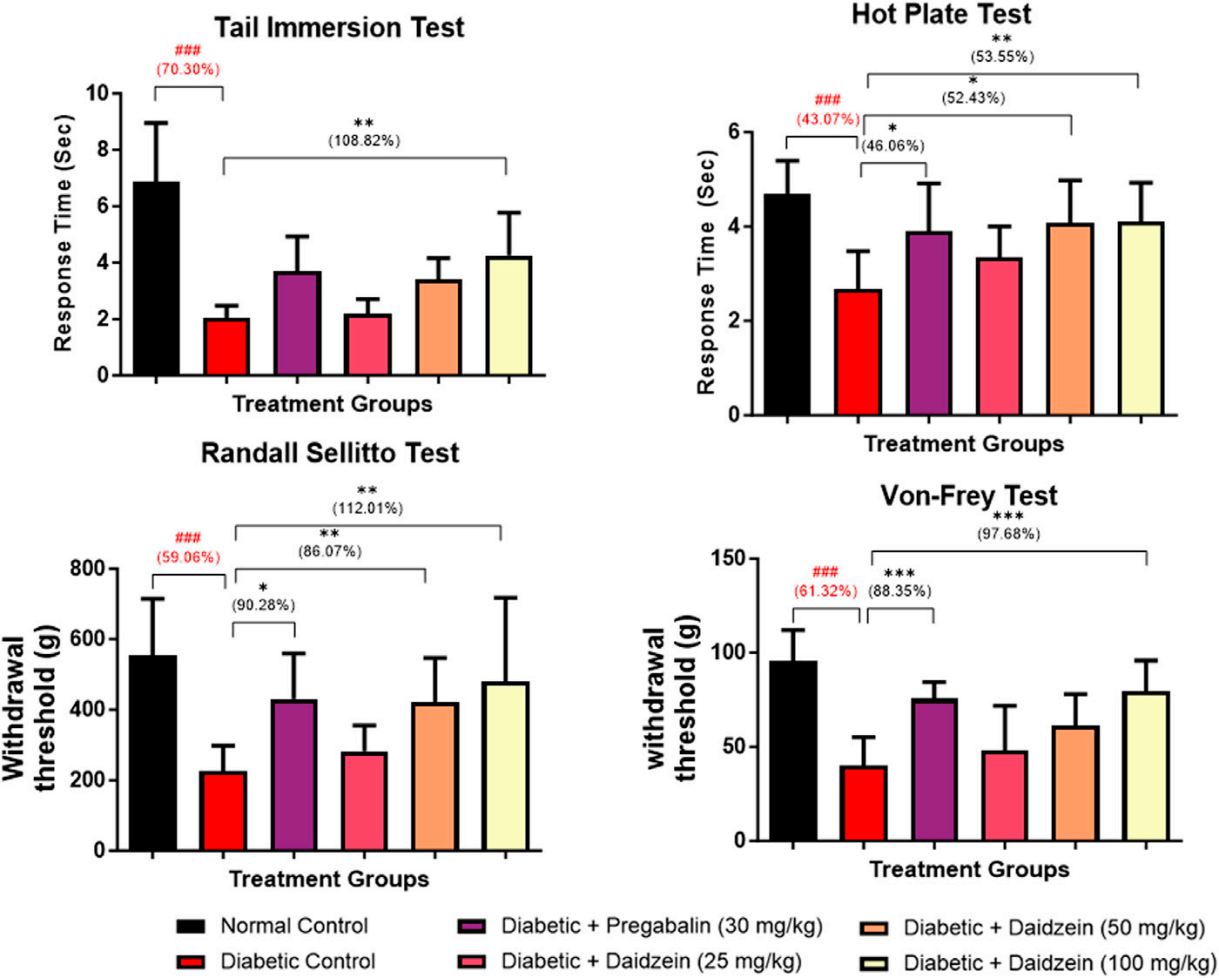
Effect of Daidzein on behavioural parameters. All values are expressed as Mean ± SD. ***p < 0.001, **p < 0.01, *p < 0.05 indicates the level of significance when treatment groups compared with the diabetic control group and ^###^ *p* < 0.001 when the diabetic control group compared with the normal control group.

Diabetic animals showed a significant decrease in paw withdrawal latency (*p* < 0.001) compared to normal control animals measured using Von-Frey filament. Treatment with Daidzein at a dose of 100 mg/kg and pregabalin (30 mg/kg) significantly improved (*p* < 0.001, *p* < 0.001) the paw withdrawal latency by 97.68% and 88.35% after 4 weeks of treatment (Figure 2).

### 4.3 Effect of daidzein on nerve conduction velocities

A significant decrease in MNCV (*p* < 0.001) was observed in diabetic control animals compared to normal control animals. Treatment with Daidzein at a dose of 50 and 100 mg/kg and pregabalin (30 mg/kg) significantly improved (*p* < 0.01, *p* < 0.001, and *p* < 0.001, respectively) the motor nerve conduction velocity after 28 days of treatment when compared with diabetic control animals.

**FIGURE 3:**
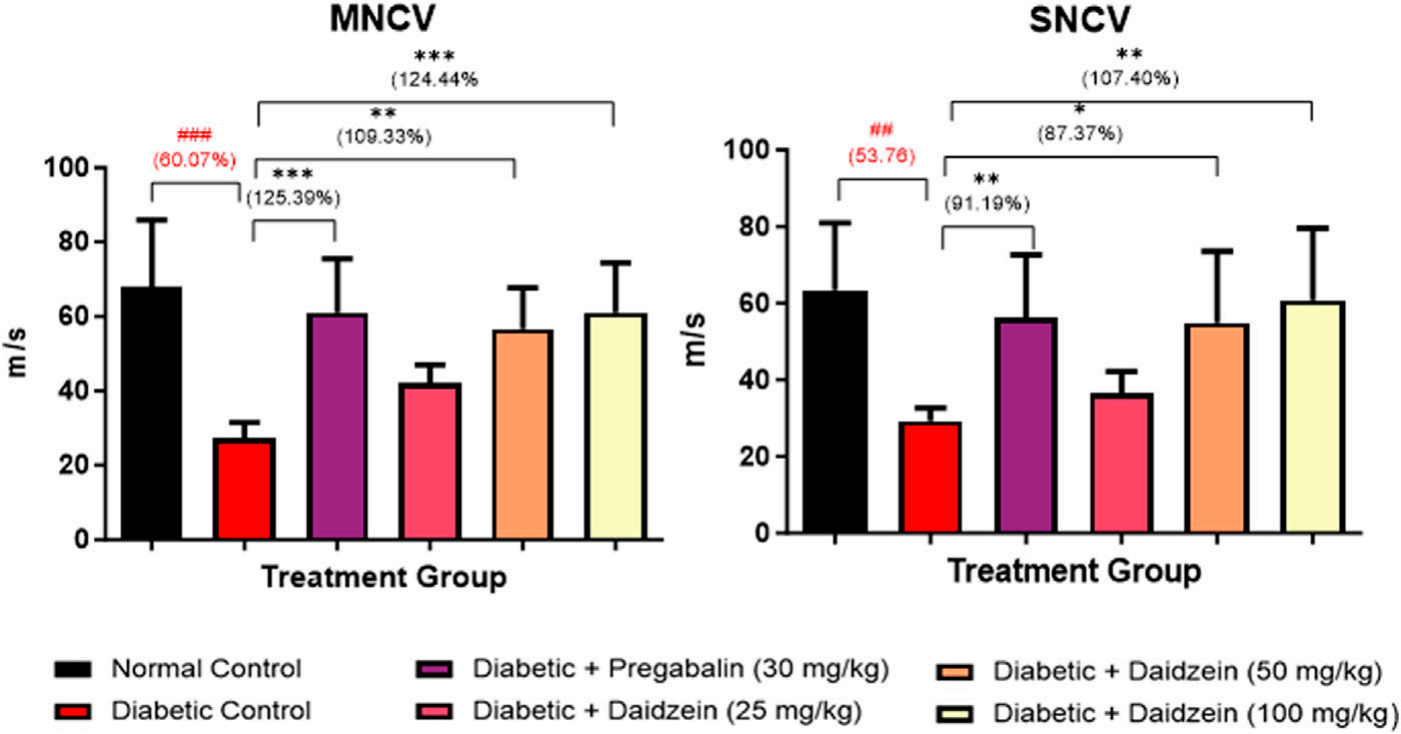
Effect of Daidzein on nerve conduction velocities. All values are expressed as Mean ± SD. ***p < 0.001, **p < 0.01, *p < 0.05 indicates the level of significance when treatment groups compared with the diabetic control group and ^###^ *p* < 0.001 when the diabetic control group compared with the normal control group.

Similarly, SNCV was also found to be decreased (*p* < 0.01) in the diabetic control group as compared to the normal control group. Treatment with Daidzein at doses of 50 and 100 mg/kg and pregabalin (30 mg/kg) significantly improved (*p* < 0.05, *p* < 0.01, and *p* < 0.01) the sensory nerve conduction velocity by 87.34%, 107.33%, and 91.19% respectively (Figure 3).

### 4.4 Effect of daidzein on sciatic nerve oxidative stress

Diabetic animals showed a significant decrease in the level of GSH, SOD, and CAT (*p* < 0.01, *p* < 0.01, and *p* < 0.001) when compared to normal control animals. Treatment with Daidzein at a dose of 100 mg/kg significantly prevented the loss of antioxidant enzymes (*p* < 0.05, *p* < 0.05, and *p* < 0.001).

MDA level was found to be significantly elevated in diabetic control animals as compared to normal control animals (*p* < 0.001). Treatment with Daidzein at a dose of 25, 50, and 100 mg/kg and pregabalin (30 mg/kg) significantly reduced the elevated level of MDA after 4 weeks of treatment (*p* < 0.05, *p* < 0.01, *p* < 0.001, and *p* < 0.001 respectively) (Table 1).

**TABLE 1:** Effect of Daidzein on oxidative stress parameters.

Parameters	Treatment groups					
	Normal control	Diabetic control	Diabetic + pregabalin (30 mg/kg)	Diabetic + daidzein (25 mg/kg)	Diabetic + daidzein (50 mg/kg)	Diabetic + daidzein (100 mg/kg
GSH (μmol/mg of protein)	19.45 ± 3.27	4.15 ± 0.59^##^	11.70 ± 2.57	11.07 ± 3.01	12.87 ± 3.95	15.77 ± 3.09*
SOD (U/mg of protein)	0.99 ± 0.16	0.29 ± 0.03^##^	0.70 ± 0.14	0.58 ± 0.14	0.71 ± 0.13	0.76 ± 0.10*
CAT (μm of H_2_0_2_ decompose/min/mg of protein)	0.375 ± 0.04	0.022 ± 0.004^###^	0.212 ± 0.024**	0.075 ± 0.026	0.25 ± 0.046***	0.319 ± 0.063***
MDA (μmol/mg of protein)	3.56 ± 0.73	10.75 ± 0.91^###^	5.66 ± 0.75***	6.88 ± 0.72*	6.48 ± 0.99**	4.23 ± 0.69***

All values are expressed as Mean ± SD. ***p < 0.001, **p < 0.01 and *p < 0.05 compared with diabetic control. ^###^ *p* < 0.001 and ^##^ *p* < 0.01 when compared with normal control.

### 4.5 Effect of daidzein on NOX-4 expression

**FIGURE 4:**
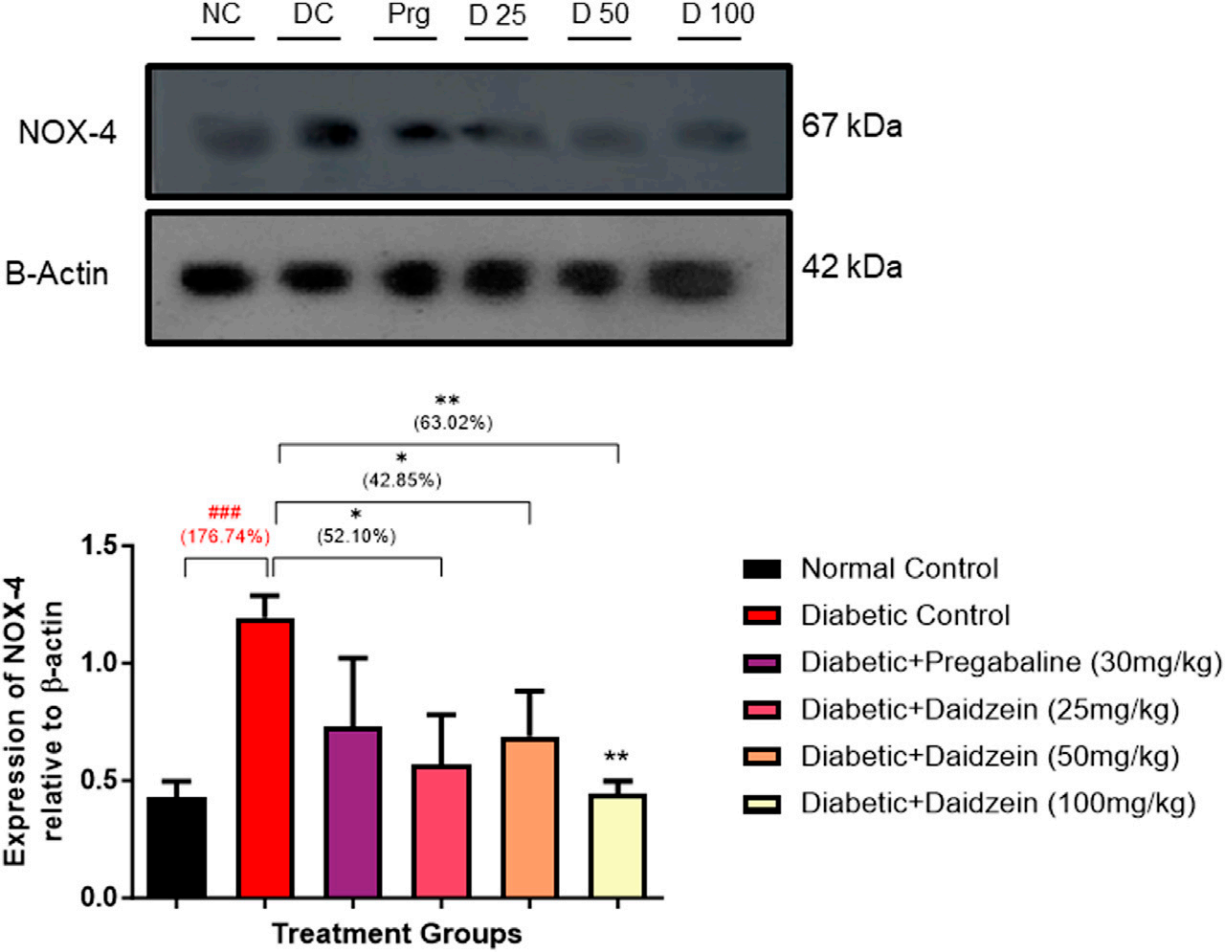
Effect of Daidzein on the relative expression of NOX-4 in sciatic nerve. All values are expressed as Mean ± SD. *p < 0.05, **p < 0.01 indicates the level of significance when treatment groups compared with the diabetic control group and ^###^ *p* < 0.001 when the diabetic group compared with the normal control group. NC, Normal Conrtol; DC, Diabetic Control; Prg- Diabetic + Pregabalin (30 mg/kg), D25-Diabetic + Daidzein (25 mg/kg), D50-Diabetic + Daidzein (50 mg/kg), D100-Diabetic + Daidzein (100 mg/kg).

Diabetic animals showed a significant increase in the expression of NOX-4 when compared to normal control animals. Treatment with Daidzein significantly reduced NOX-4 (*p* < 0.01) expression by 52.10%, 42.85%, and 63.02% respectively in the sciatic nerve (Figure 4).

## 5 Discussion

Diabetes is a metabolic condition marked by persistent and unregulated high levels of blood sugar, giving rise to various complications in both small and large blood vessels. One such complication is diabetic neuropathy. Extended periods of elevated blood sugar contribute to the heightened production of advanced glycation end products (AGEs) and their corresponding receptors (AGER). This process leads to the generation of reactive oxygen species (ROS), causing damage to nerves and disrupting their conduction system (Zhao et al., 2014).

NOX-4 is a membrane-bound enzyme that utilizes oxygen and generates reactive oxygen species during hyperglycaemia. In normal conditions, NOX-4 is present in dissociated form and becomes assembled into a functional oxidase complex upon stimulation in a hyperglycaemic state and generates superoxide free radical by transferring one electron to oxygen from NADPH. During this process, O_2_ is transported from the extracellular space to the cell interior, and the H^+^ is exported, which is the main reason behind the development of oxidative neuronal damage (Bedard and Krause, 2007). Overexpression of NOX-4 is also responsible for activating inflammatory and apoptotic mediators such as nuclear factor–kappa beta (NF-kβ) and tissue necrosis factor (TNF-α) (Laddha and Kulkarni, 2020). In addition, activation of the polyol pathway, protein kinase C (PKC), poly (ADP-ribose) polymerase pathway due to prolonged hyperglycaemia also contributes to the development of neuroinflammation and nerve damage via reactive oxygen species formation.

In diabetic neuropathy, abnormal metabolic rates and insufficient insulin secretion due to pancreatic β-cell damage impair glucose transportation into cells. Consequently, the body shifts to utilizing fat for energy, significantly reducing body weight in diabetic control animals. Daidzein, a phytoestrogen, enhances insulin sensitivity and glucose metabolism (Ubaid et al., 2023), resulting in a significant improvement in body weight in animals treated with daidzein. By preventing pancreatic β-cell damage through the inhibition of NOX-4 overexpression, daidzein also helps reduce elevated blood glucose levels (Choi et al., 2008).

Neuropathic pain linked to excessive generation of inflammatory cytokines and abnormality in nerve fiber because of endothelial dysfunction during hyperglycaemia is an essential characteristic of diabetic neuropathy. Neuronal dysfunction affects the balance between non-painful and painful stimuli by damaging the inhibitory pathway or overstimulating the nociceptive pathway, which causes pain without involving nociceptors (Dubin and Patapoutian, 2010).

Assessments of behavioural response to mechanical and thermal stimuli provide an idea about nociception, hyperalgesia and allodynia and provide valuable information regarding the mechanisms of abnormal sensation and pain associated with prolonged hyperglycaemia. ROS damage due to NOX-4 overexpression in the sciatic nerve is crucial for abnormal sensation and pain. Diabetic animals showed thermal nociception, mechanical hyperalgesia, and mechanical allodynia. Daidzein treatment inhibited nociceptive pain and reduced thermal nociception, mechanical hyperalgesia, and mechanical allodynia due to its anti-inflammatory effects mediated through NOX-4 inhibition. The inhibition of NOX-4 by daidzein decreases the production of reactive oxygen species (ROS), which are key contributors to inflammation and oxidative stress in diabetic neuropathy (Kallenborn-Gerhardt et al., 2012; Geng et al., 2015). The results of the treatment groups are comparable with pregabalin which is the first-line drug for the treatment of diabetic peripheral neuropathy. In the present study pregabalin treated group is considered a positive control group. The effect of pregabalin is also due to its anti-inflammatory potential (Sałat et al., 2013; FDA, 2019). In addition to this, impaired nerve conduction because of axonal degeneration and myelin breakdown is also one of the important observations of STZ-induced diabetic rats. Increased influx of polyol pathway leads to deposition of sorbitol in the sciatic nerve, which affects Na^+^/K^+^/ATPase activity and is the important reason for the reduction in nerve conduction. Another reason that also plays a crucial role in affecting nerve conduction by affecting axonal activity is inflammation (Raccah et al., 1998). Diabetic animals showed a significant reduction in MNCV and SNCV in the sciatic nerve. Treatment with Daidzein prevented the ROS damage to the nerve and improved the nerve conduction velocity. Improvement in the conduction velocities of daidzein mid and high-dose treatment is similar to that of standard pregabalin treatment which also possesses antioxidant properties (Sałat et al., 2013). The antioxidant defense enzymes, such as glutathione (GSH), superoxide dismutase (SOD), and catalase (CAT), were found to be reduced in the sciatic nerve of diabetic subjects, while lipid peroxidation levels were increased due to elevated ROS production from activated NOX-4 in hyperglycemic conditions. This imbalance leads to neuronal demyelination, abnormal mitochondrial function, and accumulation of extracellular matrix. Daidzein treatment mitigated these effects by preserving antioxidant enzyme levels and reducing lipid peroxidation through ROS scavenging. Additionally, daidzein’s inhibition of NOX-4 expression further protected neurons from oxidative damage. This effect of daidzein is similar to the study carried out in our laboratory against diabetic cardiomyopathy (Laddha and Kulkarni, 2021b). NOX-4 encodes an NADPH oxidase enzyme, which produces reactive oxygen species (such as H_2_O_2_) by transferring electrons from NADPH to molecular oxygen. NOX-4 is primarily regulated through transcriptional mechanisms. A recent *in vitro* study reported in the year 2019 by Yu T and co-workers showed the role of NOX-4 in the hyperglycaemia-induced apoptosis of Schwann cells which were involved in the development of diabetic peripheral neuropathy (Yu et al., 2019). We have checked the expression of NOX-4 in the sciatic nerve. Diabetic animals showed a significant increase in the expression of NOX-4, treatment with Daidzein inhibits NOX-4 expression and prevents the formation of reactive oxygen species.

In the current investigation, we observed notable intra-group variability in the Randall Selitto test, contributing to the significant enhancement in force-bearing capacity among both normal and treated animals. Moreover, a comparison between the treatment and disease control groups demonstrates over 100% improvement which does not indicate parameters are restored to normal levels. The comparison underscores its substantial impact in mitigating the effect as compared to the disease control group.

## 6 Conclusion

The results of the study indicate that Daidzein treatment mitigates oxidative stress by inhibiting NOX-4, reducing ROS production, and preserving antioxidant enzymes. This approach attenuates nerve damage, improves nerve conduction velocity, and alleviates neuropathic pain, paralleling effects seen with standard treatments like pregabalin. These findings highlight daidzein’s potential as a novel therapeutic strategy against diabetic neuropathy by targeting oxidative stress pathways.

## Data Availability

The original contributions presented in the study are included in the article/Supplementary Material, further inquiries can be directed to the corresponding author.

## References

[B1] Ängeby Möller K. Johansson B. Berge O. G. (1998). Assessing mechanical allodynia in the rat paw with a new electronic algometer. J. Neurosci. Met. 84, 41–47. 10.1016/S0165-0270(98)00083-1 9821632

[B2] Bedard K. Krause K. H. (2007). The NOX family of ROS-generating NADPH oxidases: physiology and pathophysiology. Physiol. Rev. 87, 245–313. 10.1152/physrev.00044.2005 17237347

[B3] Bhatt L. K. Veeranjaneyulu A. (2010). Minocycline with aspirin: a therapeutic approach in the treatment of diabetic neuropathy. Neurol. Sci. 31, 705–716. 10.1007/s10072-010-0243-3 20213226

[B4] Chen W. Ma X. Lin Y. Xiong Y. Zheng C. Hu Y. (2016). Dietary supplementation with a high dose of daidzein enhances the antioxidant capacity in swine muscle but experts pro-oxidant function in liver and fat tissues. J. Anim. Sci. Biotechnol. 7, 43. 10.1186/S40104-016-0102-Z 27486514 PMC4969673

[B5] Choi M. S. Jung U. J. Yeo J. Kim M. J. Lee M. K. (2008). Genistein and daidzein prevent diabetes onset by elevating insulin level and altering hepatic gluconeogenic and lipogenic enzyme activities in non-obese diabetic (NOD) mice. Diabetes Metab. Res. Rev. 24, 74–81. 10.1002/dmrr.780 17932873

[B6] Das D. Sarkar S. Bordoloi J. Wann S. B. Kalita J. Manna P. (2018). Daidzein, its effects on impaired glucose and lipid metabolism and vascular inflammation associated with type 2 diabetes. BioFactors 44, 407–417. 10.1002/biof.1439 30191623

[B7] Dewanjee S. Das S. Das A. K. Bhattacharjee N. Dihingia A. Dua T. K. (2018). Molecular mechanism of diabetic neuropathy and its pharmacotherapeutic targets. Eur. J. Pharmacol. 833, 472–523. 10.1016/j.ejphar.2018.06.034 29966615

[B8] Domingueti C. P. Dusse LMSA Carvalho M. D. G. De Sousa L. P. Gomes K. B. Fernandes A. P. (2016). Diabetes mellitus: the linkage between oxidative stress, inflammation, hypercoagulability and vascular complications. J. Diabetes Complicat. 30, 738–745. 10.1016/j.jdiacomp.2015.12.018 26781070

[B9] Dubin A. E. Patapoutian A. (2010). Nociceptors: the sensors of the pain pathway. J. Clin. Investigation 120, 3760–3772. 10.1172/JCI42843 PMC2964977 21041958

[B10] Ellman G. L. (1959). Tissue sulfhydryl groups. Arch. Biochem. Biophys. 82, 70–77. 10.1016/0003-9861(59)90090-6 13650640

[B11] Elumalai S. Karunakaran U. Moon J. S. Won K. C. (2021). NADPH oxidase (NOX) targeting in diabetes: a special emphasis on pancreatic β-cell dysfunction. Cells 10, 1573. 10.3390/CELLS10071573 34206537 PMC8307876

[B12] FDA (2019). First generic drug approvals. Silver Spring, MD: FDA. Available at: https://www.fda.gov/drugs/first-generic-drug-approvals/2019-first-generic-drug-approvals (Accessed April 2, 2024).

[B13] Geis C. Geuss E. Sommer C. Schmidt HHHW Kleinschnitz C. (2017). NOX4 is an early initiator of neuropathic pain. Exp. Neurol. 288, 94–103. 10.1016/J.EXPNEUROL.2016.11.008 27856286

[B14] Geng Z. Tong X. Jia H. (2015). Reactive oxygen species (ROS) mediates non-freezing cold injury of rat sciatic nerve. Int. J. Clin. Exp. Med. 8, 15700–15707. 26629065 PMC4658954

[B15] Hosseini A. Abdollahi M. (2013). Diabetic neuropathy and oxidative stress: therapeutic perspectives. Oxid. Med. Cell. Longev. 2013, 168039. 10.1155/2013/168039 23738033 PMC3655656

[B16] Kallenborn-Gerhardt W. Schröder K. del Turco D. Lu R. Kynast K. Kosowski J. (2012). NADPH oxidase-4 maintains neuropathic pain after peripheral nerve injury. J. Neurosci. 32, 10136–10145. 10.1523/JNEUROSCI.6227-11.2012 22836249 PMC6703722

[B17] Kayser V. (2013). “Randall-selitto paw pressure test,” in Encyclopedia of pain. Berlin, Germany: Springer Berlin Heidelberg, 3357–3360. 10.1007/978-3-642-28753-4_3726

[B18] Kim J. W. Jin Y. C. Kim Y. M. Rhie S. Kim H. J. Seo H. G. (2009). Daidzein administration *in vivo* reduces myocardial injury in a rat ischemia/reperfusion model by inhibiting NF-kappaB activation. Life Sci. 84, 227–234. 10.1016/j.lfs.2008.12.005 19109981

[B19] Laddha A. P. Kulkarni Y. A. (2020). NADPH oxidase: a membrane-bound enzyme and its inhibitors in diabetic complications. Eur. J. Pharmacol. 881, 173206. 10.1016/j.ejphar.2020.173206 32442539

[B20] Laddha A. P. Kulkarni Y. A. (2021a). Daidzein ameliorates diabetic retinopathy in experimental animals. Life Sci. 265, 118779. 10.1016/j.lfs.2020.118779 33217441

[B21] Laddha A. P. Kulkarni Y. A. (2021b). Daidzein mitigates myocardial injury in streptozotocin-induced diabetes in rats. Life Sci. 284, 119664. 10.1016/J.LFS.2021.119664 34090859

[B22] Laddha A. P. Kulkarni Y. A. (2022). Daidzein attenuates urinary bladder dysfunction in streptozotocin-induced diabetes in rats by NOX-4 and RAC-1 inhibition. Naunyn Schmiedeb. Arch. Pharmacol. 395, 975–986. 10.1007/S00210-022-02246-Y 35538367

[B23] Lowry O. H. Rosebrough N. J. Farr A. L. Randall R. J. (1951). Protein measurement with the folin phenol reagent. J. Biol. Chem. 193, 265–275. 10.1016/S0021-9258(19)52451-6 14907713

[B24] Nasiry D. Khalatbary A. R. Ahmadvand H. Talebpour Amiri F. Akbari E. (2017). Protective effects of methanolic extract of Juglans regia L. leaf on streptozotocin-induced diabetic peripheral neuropathy in rats. BMC Complement. Altern. Med. 17, 476. 10.1186/s12906-017-1983-x 28969623 PMC5625610

[B25] Ohkawa H. Ohishi N. Yagi K. (1979). Assay for lipid peroxides in animal tissues by thiobarbituric acid reaction. Anal. Biochem. 95, 351–358. 10.1016/0003-2697(79)90738-3 36810

[B26] Oza M. J. Kulkarni Y. A. (2020). Formononetin ameliorates diabetic neuropathy by increasing expression of SIRT1 and NGF. Chem. Biodivers. 17 (6), e2000162. 10.1002/CBDV.202000162 32459048

[B27] Paoletti F. Mocali A. Aldinucci D. (1990). Superoxide-driven NAD(P)H oxidation induced by EDTA-manganese complex and mercaptoethanol. Chem. Biol. Interact. 76, 3–18. 10.1016/0009-2797(90)90030-Q 2168295

[B28] Peng Y. Shi Y. Zhang H. Mine Y. Tsao R. (2017). Anti-inflammatory and anti-oxidative activities of daidzein and its sulfonic acid ester derivatives. J. Funct. Foods 35, 635–640. 10.1016/j.jff.2017.06.027

[B29] Raccah D. Coste T. Cameron N. E. Dufayet D. Vague P. Hohman T. C. (1998). Effect of the aldose reductase inhibitor tolrestat on nerve conduction velocity, Na/K ATPase activity, and polyols in red blood cells, sciatic nerve, kidney cortex, and kidney medulla of diabetic rats. J. Diabetes Complicat. 12, 154–162. 10.1016/S1056-8727(97)00093-7 9618071

[B30] Rayegan S. Dehpour A. R. Sharifi A. M. (2017). Studying neuroprotective effect of atorvastatin as a small molecule drug on high glucose-induced neurotoxicity in undifferentiated PC12 cells: role of NADPH oxidase. Metab. Brain Dis. 32, 41–49. 10.1007/s11011-016-9883-1 27476541 PMC7102122

[B31] Sałat K. Librowski T. Nawiesniak B. Gluch-Lutwin M. (2013). Evaluation of analgesic, antioxidant, cytotoxic and metabolic effects of pregabalin for the use in neuropathic pain. Neurol. Res. 35, 948–958. 10.1179/1743132813Y.0000000236 23816319

[B32] Schreiber A. K. Nones C. F. Reis R. C. Chichorro J. G. Cunha J. M. (2015). Diabetic neuropathic pain: physiopathology and treatment. World J. Diabetes 6, 432–444. 10.4239/wjd.v6.i3.432 25897354 PMC4398900

[B33] Suryavanshi S. V. Kulkarni Y. A. (2020). Escin alleviates peripheral neuropathy in streptozotocin induced diabetes in rats. Life Sci. 254, 117777. 10.1016/J.LFS.2020.117777 32407839

[B34] Ubaid M. Salauddin N. Shadani M. A. Kawish S. M. Albratty M. Makeen H. A. (2023). Daidzein from dietary supplement to a drug candidate: an evaluation of potential. ACS Omega 8, 32271–32293. 10.1021/ACSOMEGA.3C03741 37780202 PMC10538961

[B35] Volpe C. M. O. Villar-Delfino P. H. Dos Anjos P. M. F. Nogueira-Machado J. A. (2018). Cellular death, reactive oxygen species (ROS) and diabetic complications. Cell. Death Dis. 9, 119. 10.1038/s41419-017-0135-z 29371661 PMC5833737

[B36] Wei J. Yang F. Gong C. Shi X. Wang G. (2019). Protective effect of daidzein against streptozotocin-induced Alzheimer’s disease via improving cognitive dysfunction and oxidative stress in rat model. J. Biochem. Mol. Toxicol. 33, 22319. 10.1002/jbt.22319 30897277

[B37] Yu T. Xin Q. Xu F. Li L. (2019). Research on the mechanism of high glucose affecting the apoptosis of schwann cells by Nox4 NADPH oxidase. Zhongguo Ying Yong Sheng Li Xue Za Zhi 35, 130–134. 10.12047/j.cjap.5719.2019.029 31250603

[B38] Zhao W. C. Zhang B. Liao M. J. Zhang W. X. He W. Y. Wang H. B. (2014). Curcumin ameliorated diabetic neuropathy partially by inhibition of NADPH oxidase mediating oxidative stress in the spinal cord. Neurosci. Lett. 560, 81–85. 10.1016/j.neulet.2013.12.019 24370596

[B39] Zheng W. Sun R. Yang L. Zeng X. Xue Y. An R. (2017). Daidzein inhibits choriocarcinoma proliferation by arresting cell cycle at G1 phase through suppressing ERK pathway *in vitro* and *in vivo* . Oncol. Rep. 38, 2518–2524. 10.3892/or.2017.5928 28849226

